# Swimming With Sharks: Left Main Coronary Obstruction Following Transcatheter Aortic Valve Implantation

**DOI:** 10.7759/cureus.40514

**Published:** 2023-06-16

**Authors:** Nelson Barrera, Francisco Gallegos, Salomon Chamay, Roberto Cerrud-Rodriguez

**Affiliations:** 1 Division of Internal Medicine, St. Barnabas Hospital Health System, Bronx, USA; 2 Division of Cardiology, Montefiore Medical Center, Albert Einstein College of Medicine, Bronx, USA

**Keywords:** sinotubular junction, aortic stenosis (as), delayed coronary obstruction in tavr, coronary artery occlusion, trans catheter aortic valve implantation (tavi)

## Abstract

Delayed coronary obstruction (DCO) occurs when there is obstruction of the coronary ostia following a transcatheter aortic valvular implantation (TAVI). It is an uncommon but serious complication that often leads to death, usually presents as severe hypotension after TAVI, and should be suspected if migration of the valve occurs. We report the case of a 70-year-old female patient with severe aortic stenosis who underwent TAVI using a 26-mm CoreValve Evolut Pro (Medtronic, Dublin, Ireland). Although the valve was implanted successfully, she experienced hypotension with intermittent ST elevations and had a cardiac arrest shortly after, requiring Advanced Cardiovascular Life Support (ACLS). An aortogram showed sealing of the sinotubular junction (STJ) by CoreValve, without coronary flow. CoreValve was then snared and repositioned in the ascending aorta recovering coronary flow and cardiac pulsatility. A second TAVI was performed and an Edwards 20 mm Sapiens 3 valve (Edwards Lifesciences, Irvine, CA, USA) was implanted as standard procedure.

## Introduction

Transcatheter aortic valvular implantation (TAVI) is considered a mainstream interventional procedure for patients with severe aortic valve stenosis, in either symptomatic patients or those who are asymptomatic but have a left ventricular ejection fraction less than 50%. It is also an option for those who are considered inoperable or have a high-to-moderate surgical risk [1,2]. It is associated with a lower risk of all-cause mortality and cardiovascular death in one year when compared with surgery in low-surgical-risk patients [3].

Delayed coronary obstruction (DCO) is a rare complication of TAVI, occurs in less than 1% of cases, and is associated with a high mortality rate of around 50%. Even after successful rescue with percutaneous coronary intervention, the mortality rate is approximately 20%. DCO occurs after the TAVI procedure has concluded and can be classified as early or late when it develops within seven days following TAVI or after seven days, respectively [4]. The most common presentation of early DCO is severe hypotension with cardiac arrest after valve implantation, which often requires venous-arterial extracorporeal membrane oxygenation (VA-ECMO) [5].

## Case presentation

We present a female patient in her mid-seventies with a history of transitory ischemic attack, obstructive sleep apnea, and multiple sclerosis, who was referred to the multidisciplinary structural heart disease clinic for evaluation of severe aortic stenosis. The patient was experiencing angina on exertion when climbing stairs or walking uphill (Canadian Cardiovascular Society Class II, slight limitation of ordinary activities). The physical examination revealed a 4/6 systolic murmur in the aortic area. Given the high risk of surgical aortic valve replacement, the patient was scheduled for TAVI.

The patient underwent preoperative evaluation, which included a transthoracic echocardiogram (TTE) that showed a normal ejection fraction, severe aortic valve calcification, a mean transaortic gradient of 49.67 mmHg, a peak gradient of 103.91 mmHg, and an aortic valve area of 1.2 cm^2^. A left heart catheterization was performed by the referring cardiologist and showed nonobstructive coronary artery disease.

Pre-TAVI contrast computed tomography showed a moderately calcified tricuspid aortic valve with an annular area of 6.09 mm^2^ and a perimeter of 65 mm at 35% of the R-R interval. The left coronary artery (LCA) and the right coronary arteries (RCAs) arose at 14 mm from the aortic annulus, in a right-dominant system. The diameter of the sinus of Valsalva (from sinus to commissure) was 25 mm for the right, 27 mm for the left, and 26 mm for the noncoronary sinus.

The patient was taken to the catheterization lab and was prepped in the usual sterile fashion, under monitored anesthesia. A temporary transvenous pacer (TVP) was successfully advanced to the right ventricle via the right femoral vein. Embolic protection with a SENTINEL device (Boston Scientific, Marlborough, MA, USA) was used.

A 26-mm CoreValve Evolut Pro (Medtronic, Dublin, Ireland) was introduced through the right common femoral artery. Once engagement occurred, an aortogram was performed to confirm the proper position. The valve was then partially unsheathed to the 2/3 position, and its satisfactory position and function were assessed and confirmed through aortography. The valve was then fully unsheathed and disengaged from the delivery catheter. Upon disengagement, the valve shifted upward toward the aorta; however, both echocardiography and aortography showed good coronary flow with transprosthetic gradients of less than 10 mmHg. No changes were observed in the electrocardiogram.

The procedure was deemed successful, and the SENTINEL device, arterial catheters, and sheaths were removed from the patient, leaving the femoral TVP in place as is standard practice in our institution. Approximately 10 minutes after concluding the procedure, the patient became hypotensive, and intermittent ST elevations were detected in the cardiac monitor. Despite providing inotropic support, the patient went into ventricular fibrillation, and Advanced Cardiovascular Life Support (ACLS) protocol was promptly started and continued for 5 minutes.

Left femoral arterial and venous accesses were obtained and VA-ECMO was initiated through the left femoral access. The patient was successfully defibrillated back into normal sinus rhythm. Access to the right common femoral artery was also obtained. Efforts to selectively angiograph the LCA were unsuccessful, and an aortogram revealed that the displaced CoreValve had sealed the STJ and blocked coronary flow (Video 1). The CoreValve was then snared through the right common femoral artery access using an ensnare and repositioned in the ascending aorta, restoring coronary flow (Video 1). At this stage, the patient became stabilized with the return of cardiac pulsation.

**Video 1:** (Part 1) Coronary angiography demonstrating lack of flow into the left coronary artery; (Part 2) snaring of CoreValve (Medtronic, Dublin, Ireland) and repositioning into the ascending aorta.

A multidisciplinary discussion was held regarding a second TAVR vs. open-heart surgery. Given her high risk, a decision was made to proceed with a second TAVR using an Edwards 20 mm Sapiens 3 Ultra valve (Edwards Lifesciences, Irvine, CA, USA) as the team believed that a second CoreValve may not achieve full expansion. CoreValve was then snared again, this time from the right radial artery, to avoid downward displacement when crossing with the new Sapiens 3 valve.

The Sapiens 3 valve was subsequently implanted using standard procedure. Imaging and hemodynamics confirmed the correct valve position and function. Selective coronary angiography was performed, which confirmed the patency of both the LCA and RCA (Video 2). Shortly afterward, the patient remained hemodynamically stable and required minimal inotropic support, and transesophageal echocardiography (TEE) confirmed full recovery of left ventricular function, so a decision was made to decanulate her from ECMO in the catheterization suite. She was then admitted to the critical care unit (CCU) and was discharged home six days later.

**Video 2:** Selective coronary angiography confirming patency of both the left and right coronary arteries.

The patient was seen in the TAVR clinic as an outpatient, with complaints of light memory loss and some limitations in her activity level that seemed to be steadily improving. Transthoracic echocardiography (TTE) demonstrated a normal left ventricular ejection fraction and normal prosthetic valve function.

## Discussion

Common complications of TAVR include moderate to severe paravalvular leakage, major vascular and bleeding complications, disabling stroke, acute kidney injury, and conduction abnormalities such as high-degree ventricular block. Unexplained postprocedural hypotension should raise concern for an acute tamponade due to ventricular rupture or secondary to TVP displacement. A mild-to-moderate paravalvular leak is a rare cause of shock and is generally well tolerated by patients. Hypotension after balloon dilation or valve expansion should promptly alarm physicians of possible annular or aortic root dissection or hematoma [6].

Coronary obstruction is an infrequent complication of TAVR and DCO is even rarer, with an incidence of less than 1%. Risk factors for coronary obstruction following TAVR include female sex, a previous prosthetic aortic valve in situ, a distance of <10 mm between the coronary ostia and the aortic annulus, and <28 mm diameter at the sinuses of Valsalva [5,7-8]. A systematic review of clinical outcomes of coronary occlusion following TAVR found that women comprised 81% of cases, 80% of events involved the left main coronary artery, 60% were caused by a displaced native valve leaflet, and 88% occurred within one hour of implantation [9].

The etiology of early DCO (<7 days after TAVR) is hypothesized to occur secondary to the continued expansion of the implanted valve, dissection, or an expanding hematoma leading to obstruction. In contrast, thrombus or valve stent endothelialization may cause late DCO. Percutaneous coronary intervention (PCI) is considered the preferred treatment for coronary obstruction after TAVR [7,9].

**Figure 1:**
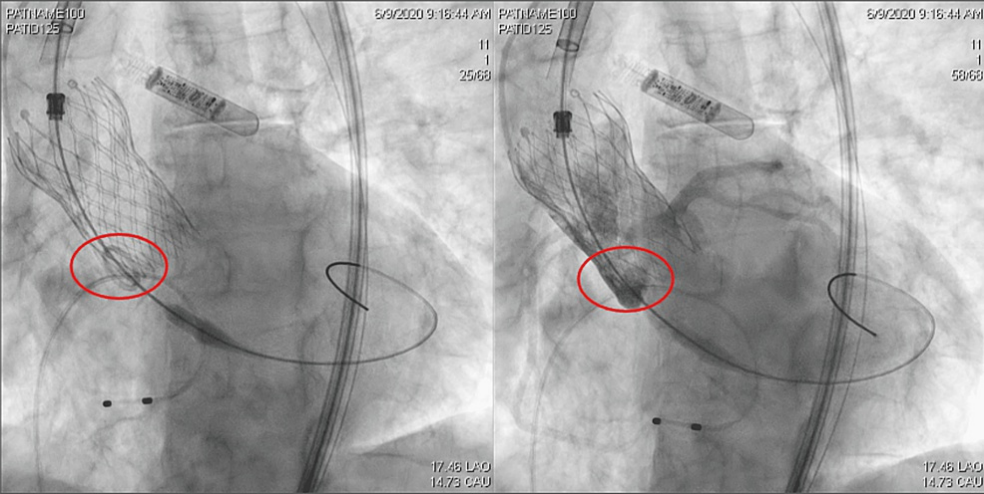
Pigtail used for the aortograms visualized below the level of the neosinuses and coronary flow occurring during systole (red circles).

In our patient’s case, a careful review of the catheterization films after the event showed that immediately post-implant, the pigtail used for the aortograms was below the level of the neosinuses and that coronary flow occurred during systole (Figure 1), which should have alerted us to the impending complication. The reason for the displacement of the first valve was unclear. However, this case highlights the importance of promptly recognizing potential complications following TAVR, specifically DCO, and acting on time.

The management of the coronary obstruction depends on the implanted prosthetic valve: CoreValve can be ensnared and repositioned, whereas Sapien 3 will need immediate cannulation of the occluded ostium using a high-pressure balloon [10].

## Conclusions

DCO following transcatheter aortic valve repair is a rare, yet potentially catastrophic complication that requires early diagnosis and prompt decision-making. The complexity of this presentation calls for a multidisciplinary team comprising interventional cardiologists, cardiothoracic surgeons, and an ECMO team to optimize the chances of survival. It is crucial to note that the management of DCO varies based on the type of valve implanted. For instance, ensnaring and repositioning techniques are appropriate for CoreValve, while the Sapiens 3 valve requires PCI using a high-pressure balloon to address the occluded ostium.
